# The Therapeutic Potential of AN-7, a Novel Histone Deacetylase Inhibitor, for Treatment of Mycosis Fungoides/Sezary Syndrome Alone or with Doxorubicin

**DOI:** 10.1371/journal.pone.0146115

**Published:** 2016-01-11

**Authors:** Lilach Moyal, Nataly Feldbaum, Neta Goldfeiz, Ada Rephaeli, Abraham Nudelman, Michal Weitman, Nataly Tarasenko, Batia Gorovitz, Leah Maron, Shiran Yehezkel, Iris Amitay-Laish, Ido Lubin, Emmilia Hodak

**Affiliations:** 1 Laboratory for Molecular Dermatology, Felsenstein Medical Research Center, Petach Tikva, and Sackler Faculty of Medicine, Tel Aviv University, Tel Aviv, Israel; 2 Department of Dermatology, Rabin Medical Center, Petach Tikva, and Sackler Faculty of Medicine, Tel Aviv University, Tel Aviv, Israel; 3 Laboratory for Pharmacology and Experimental Oncology, Felsenstein Medical Research Center, Petach Tikva, and Sackler Faculty of Medicine, Tel Aviv University, Tel Aviv, Israel; 4 Division of Medicinal Chemistry, Department of Chemistry, Bar Ilan University, Ramat Gan, Israel; 5 Core Facility, Felsenstein Medical Research Center, Petach Tikva, and Sackler Faculty of Medicine, Tel Aviv University, Tel Aviv, Israel; Broad Institute of Harvard and MIT, UNITED STATES

## Abstract

The 2 histone deacetylase inhibitors (HDACIs) approved for the treatment of cutaneous T-cell lymphoma (CTCL) including mycosis fungoides/sezary syndrome (MF/SS), suberoylanilide hydroxamic acid (SAHA) and romidepsin, are associated with low rates of overall response and high rates of adverse effects. Data regarding combination treatments with HDACIs is sparse. Butyroyloxymethyl diethylphosphate (AN-7) is a novel HDACI, which was found to have selective anticancer activity in several cell lines and animal models. The aim of this study was to compare the anticancer effects of AN-7 and SAHA, either alone or combined with doxorubicin, on MF/SS cell lines and peripheral blood lymphocytes (PBL) from patients with Sezary syndrome (SPBL). MyLa cells, Hut78 cells, SPBL, and PBL from healthy normal individuals (NPBL) were exposed to the test drugs, and the findings were analyzed by a viability assay, an apoptosis assay, and Western blot. AN-7 was more selectively toxic to MyLa cells, Hut78 cells, and SPBL (relative to NPBL) than SAHA and also acted more rapidly. Both drugs induced apoptosis in MF/SS cell lines, SAHA had a greater effect on MyLa cell line, while AN-7 induced greater apoptosis in SPBL; both caused an accumulation of acetylated histone H_3_, but AN-7 was associated with earlier kinetics; and both caused a downregulation of the HDAC1 protein in MF/SS cell lines. AN-7 acted synergistically with doxorubicin in both MF/SS cell lines and SPBL, and antagonistically with doxorubicin in NPBL. By contrast, SAHA acted antagonistically with doxorubicin on MF/SS cell lines, SPBL, and NPBL, leaving <50% viable cells. In conclusion, AN-7 holds promise as a therapeutic agent in MF/SS and has several advantages over SAHA. Our data provide a rationale for combining AN-7, but not SAHA, with doxorubicin to induce the cell death in MF/SS.

## Introduction

Mycosis fungoides (MF), the most common type of cutaneous T-cell lymphoma (CTCL), is manifested clinically by patches that may gradually develop into plaques and eventually tumors [1,2]. Sézary syndrome (SS) is a rare aggressive leukemic-phase type of MF [3]. There is no known cure for MF/SS. Skin-directed therapy is the key to management of early-stage MF, and systemic therapy is essential in advanced MF and in cases of SS. Although there are several systemic therapeutic options primarily for advanced MF and SS for slowing disease progression and preserving quality of life as long as possible, response rates are relatively low [4,5]. Therefore, novel effective treatments tailored for advanced-stage MF and SS and recurrent/refractory early-stage MF are required.

Histone deacetylase inhibitors (HDACIs) have been found to induce cell death in both solid and hematological malignancies [6], either extrinsically (death receptor pathway) or intrinsically (caspase activation, mitochondrial pathway), via transcription-dependent and transcription-independent mechanisms [7, 8]. Suberoylanilide hydroxamic acid, (SAHA, vorinostat), approved by the US Food and Drug Administration (FDA) in 2006 for the treatment of CTCL, is an orally bioavailable HDACI of classes I, II, and V [9,10]. It induces accumulation of acetylated histones, cell-cycle arrest, and apoptosis selectively in cancer cell lines [11]. Depsipeptide (Romidepsin) was the second HDACI approved by the FDA in 2009 for CTCL [12]. These HDACIs, given as a single agent, yield overall response rate of 30–35%, but a complete response rate of only 2–6% [13]. Given the limited clinical efficacy of these two HDACIs and their high rates of adverse effects, there is an ongoing effort to develop new HDACIs with improved efficacy and selectivity. Combination therapy may yield benefits from potentiating the efficacy of HDACI with other agents [14,15]. However, currently data regarding combination treatments is strikingly sparse [16–18].

Prompted by findings that HDACIs sensitize tumor lines to DNA-damage inducers [19,20], it has been suggested that combining HDACIs with anti-tumor agents such as doxorubicin (Dox), a widely used anthracycline derivative, may yield better clinical results. Dox acts via formaldehyde-mediated alkylation of DNA with consequent adduct formation [21], and have shown high effectiveness against a broad range of cancers. Clinical studies with the HDACI-Dox combination treatment have reported promising results in various types of cancer, but data specifically for CTCL remain sparse [22,23].

Butyroyloxymethyl diethyl phosphate (AN-7) is a novel HDACI, which is a water-soluble, orally active prodrug of the HDACI butyric acid. Upon hydrolytic degradation, it releases butyric acid, formaldehyde, and phosphoric acid. Like other derivatives of butyric acid, AN-7 inhibits HDAC classes I and II and was found to exert anticancer activities *in vitro* [24–30] and *in vivo*, in mouse model [24,26]. We have previously shown that AN-7 is a selective anti-cancer drug displaying preferential cytotoxicity against leukemic and glioblastoma cells compared to their normal cellular counterparts-normal mononuclear and astrocytes cells [27]. Furthermore, AN-7 was shown to exhibit selective toxic and apoptotic effect in murine mammary 4T1, and human glioblastoma U251 cancer cell lines, as compared to neonatal rat cardiomyocytes, cardiofibroblasts and astrocytes [30]. Moreover it interacts synergistically with Dox in mice bearing mammary tumors [29] and in the MCF-7 cell line [25].

The aim of the present study was to evaluate the anti-MF/SS effect of AN-7; we studied the anticancer effect of AN-7 on MF/SS cell lines and PBL of SS patients, either alone or combined with Dox, and in comparison with SAHA.

## Materials and Methods

All patients provided their written informed consent to participate in this study, approved by the Ethics Committee of Rabin Medical Center (Ref. 6515 for PBL from healthy subjects and ref. 7175 for PBL from patients with SS).

### Compounds and reagents

AN-7 was synthesized as described previously [24]. The following are commercial products: SAHA, Sigma-Aldrich (Rehovot, Israel), doxorubicin hydrochloride, Teva (Petach Tikva, Israel); lymphoprep, Axis Shield (Oslo, Norway); phytohemagglutinin (PHA), Becton Dickinson (Franklin Lakes, NJ, USA); thiazolyl blue tetrazolium bromide (MTT) reagent, Sigma-Aldrich (Rehovot, Israel); fluorescein isothiocyanate-conjugated annexin V, eBioscience (San Diego, DA, USA); propidium iodide (PI), eBioscience and trypan Blue, Bio-Basic (Unionville, Canada).

### Cell lines

MyLa 2059 cells, derived from a plaque of a patient with MF [31], and Hut78 cells, derived from peripheral lymphocytes of a patient with SS [32], were generously donated by Robert Gniadecki, MD, from Copenhagen University, Copenhagen, Denmark (in June 2011).

### Peripheral blood lymphocytes

SPBL were derived from 4 patients with SS, who attended the Department of Dermatology, at Rabin Medical Center. All were treatment-naïve patients, and all were diagnosed according to the criteria of the European Organization for Research and Treatment of Cancer (EORTC)-World Health Organization (WHO) [33]. In addition, blood samples enriched with NPBL were obtained from leftover blood of 8 healthy blood donors at Magen David Adom, Sheba Medical Center, Israel.

### Isolation of human peripheral blood lymphocytes

Peripheral blood was diluted 1:3 in sterile phosphate-buffered saline (PBS). Lymphoprep was added in the same blood volume with a Pasteur pipette, and the sample was centrifuged. PBL were collected from the white median interphase, rinsed twice with PBS, and suspended in RPMI medium with 10mM HEPES to 2×10^6^ cells/mL.

#### Mossman's tetrazole test (MTT)-based viability assay

MyLa cells (10^4^), Hut78 cells (5x10^3^), SPBL and NPBL (10^5^) were seeded in triplicate in 96-well plates. The PBL were activated with PHA 40 μg/10^6^ cell for 24 h before the experiment. Drugs were added to each well as follows: AN-7, SAHA, Dox, AN-7+Dox, SAHA+Dox. The cells were then placed in a humidified incubator for 72 h. The ratios of HDACI to Dox in the combined-treatment experiments were based on the ratio of the IC_50_ of each drug alone. The MTT reagent was added to a final concentration of 0.5 mg/mL, and the cells were incubated for an additional 4 h. Thereafter, 0.1N HCl in isopropanol was added, and cell viability was determined using an ELISA reader (PowerWaveX, BioTek, Winooski, VT, USA) at a 570 nM wavelength with background subtraction at 630–690 nM.

### Trypan-blue-based viability assay

MyLa cells (2×10^5^ cells/mL), Hut78 cells (2×10^5^ cells/mL), or NPBL (1×10^6^ cells/mL (after overnight incubation with PHA) were treated with an HDACI under two conditions: long exposure—24 h for MyLa cells and Hut78 cells and 48 h for PBL; or short exposure—4 h incubation followed by washout and re-incubation with new medium for another 44 h (MyLa) or 20 h (Hut78). All samples were diluted 1:5 with trypan blue (0.5%), and unstained (viable) cells were counted under a light microscope.

#### FACS analysis with annexin V and propidium iodide staining

MyLa and Hut78 cells (2x10^5^ cells/mL) were exposed to SAHA or AN-7 as in the trypan-blue assay. The cells (2.5x10^6^ cells/mL) were washed in PBS and binding buffer and were resuspended in binding buffer, and of fluorescein isothiocyanate-conjugated annexin V (5 μL) was added to a 100 μL cell suspension for 10–15 min. Incubation was performed at room temperature under light-protected conditions. The cells were then washed in binding buffer and were resuspended in the binding buffer and propidium iodide (PI) (5 μL) was added. The samples were analyzed by flow cytometry (FACS Calibur 4.1.6, Becton Dickinson): fluorescein-labeled annexin V at a 530 nm wavelength, and PI at a 585 nm wavelength. The percentage of cells was calculated by their distribution in a fluorescence dot plot generated with FCS Express 4 software (De Novo Software, Los Angeles, CA, USA). Early (annexin V-positive) and late (annexin V+PI-positive) apoptotic cells were summed to yield the total number of apoptotic cells.

### Nuclear fractionation for histone detection

Cells (5×10^6^) were suspended in 300 μL of cytoplasmic buffer (HEPES 10 mM, KCl 10 mM, EDTA 1 mM, EGTA 1 mM, DTT 1 mM). After 20 min of incubation on ice, the mixture was passed 5 times through a 25-G syringe and then centrifuged briefly to obtain the cytoplasmic supernatant. The nuclear pellet was suspended in 40–60 μL of nuclear buffer (cytoplasmic buffer+10% glycerol), incubated with shaking at 4°C for 15 min, and centrifuged. The supernatant was collected as a nuclear fraction.

### Western blot analysis

Cell extracts were separated by SDS-polyacrylamide gel electrophoresis (SDS-PAGE), transferred to a nitrocellulose membrane, and subjected to immunoblot with primary and secondary antibodies, as listed in Table 1.

**Table 1:** Primary and Secondary Antibodies Used for Western Blot (WB).

Type	Reactivity (isotype)	Host	Dilution for WB	Manufacturer
Primary/ monoclonal	Anti-cleaved caspase-3 (IgG)	Rabbit	1:1000	Cell-signaling
Primary/ polyclonal	Anti-PARP-Poly-ADP-ribose polymerase 3 (IgG)	Rabbit	1:1000	Cell-signaling
Primary/ polyclonal	Anti-p21- cyclin-dependent kinase inhibitor 1A (IgG)	Rabbit	1:200	Santa Cruz
Primary/ polyclonal	Anti-Bax-BCL2-associated X protein (IgG)	Rabbit	1:1000	Abcam
Primary/ polyclonal	Anti-acetylated N-terminus of histone H3 (IgG)	Rabbit	1:500	Millipore
Primary/ polyclonal	Anti- histone deacetylase 1 (HDAC1) (IgG)	Rabbit	1:2000	Sigma
Primary/ monoclonal	Anti-actin (IgG)	Mouse	1:8000	Molecular probe
Secondary/polyclonal	Anti-rabbit IgG (H+L)	Goat	1:5000	LI-COR Biosciences
Secondary/polyclonal	Anti-mouse IgG (H+L)	Goat	1:5000	LI-COR Biosciences

### Computational and statistical analysis

Viability and apoptosis curves were based on the averages of at least 3 independent experiments. The standard error (SE) was calculated for each group as follows: SE = standard deviation/√n, where n is number of values in the group. The average drug concentrations causing a 50% reduction in cell viability, IC_50_, were determined with the formula for linear or polynomial regression derived from the best-fitted curve of percent viability versus drug concentrations (≥3 independent dose-response titrations). The selective toxicity index (SI) was calculated as follows: SI = IC_50_ NPBL/ IC_50_ MF/SS cells, where SI>1 indicates toxic selectivity to MF/SS cell lines, SI<1 indicates toxic selectivity to NPBL, and SI = 1 indicates no selectivity. Significant differences in selectivity among groups were analyzed by comparing the IC_50_ values using two-tailed unpaired t- test using Excel or by comparing the differential effects for all the dose response titrations using ANOVA with repeated measures.

For analysis of drug interactions, drug concentration-dependence plots for each drug alone and in combination were formulated, and the combination index (CI) was calculated using CompuSyn software (ComboSyn, Inc. Paramus, NJ, USA) [34], where CI>1 indicates an antagonist interaction between two drugs, CI = 1 indicates an additive interaction, and CI<1 a synergistic interaction.

## Results

### AN-7 is more effective and selective in MF/SS cell lines and SPBL than SAHA

**Fig 1:**
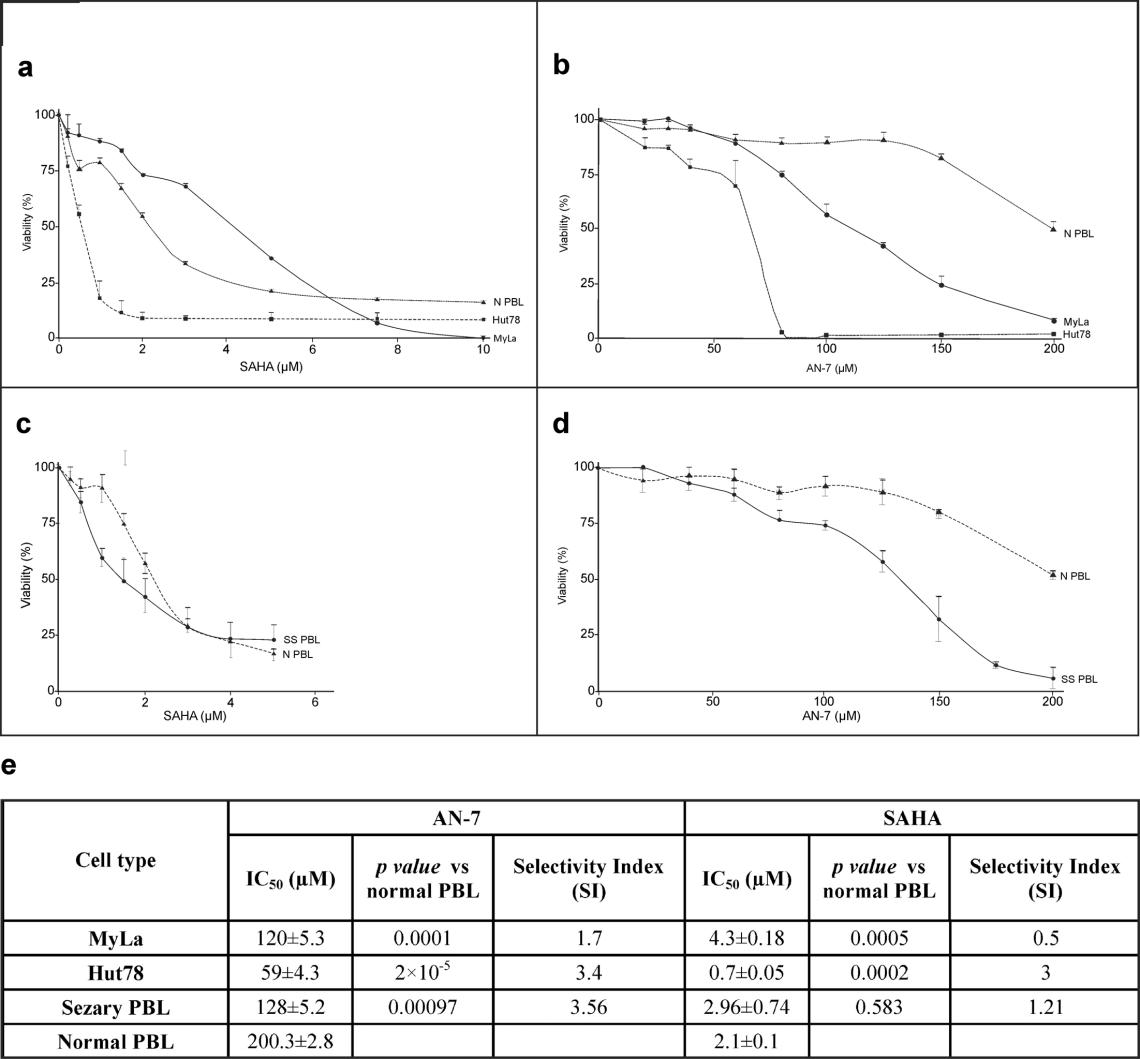
Effect of SAHA and AN-7 on the viability of MF/SS cell lines, SPBL and NPBL. Viability curves based on the MTT assay of MyLa cells, Hut78 cells, (a,b), and SPBL (n = 3) (c,d) compared to NPBL (n = 8) following treatment with SAHA (a,c) and AN-7 (b,d) for 72 h. Also shown are the IC_50_ and SI values of SAHA and AN-7 in MF/SS cell lines and SPBL and NPBL based on viability curves a-d, and their p values (e).

Dose-effect viability curves derived from the MTT-based assay showed that SAHA and AN-7 were toxic to both MyLa cells and Hut78 cells (Fig 1A and 1B). Comparison by dose-response titration (ANOVA with repeated measures) showed that SAHA was significantly selective to Hut78 cells (*p* = 1.7x10^-5^) and significantly nonselective to MyLa cells (*p* = 0.168) whereas AN-7 was significantly selective to both cell types (*p* = 1x10^-5^ and *p* = 2.89x10^-4^, respectively) (Fig 1E). Comparison by IC_50_ values yielded similar results. In the presence of high doses of AN-7, which were lethal to Hut78 and MyLa cells, 50% of the NPBL survived. By contrast, high doses of SAHA were lethal to all cells (Fig 1A and 1B).

To confirm the *in vitro* results, we tested the toxicity and selectivity of AN-7 and SAHA in SPBL (Fig 1C and 1D). Analysis by the IC_50_ values derived from the viability curves showed that AN-7 induced selective death but SAHA induced nonselective death. Results were similar on comparison by dose-response titration (*p*<0.001 and *p* = 0.5173, respectively). High doses of AN-7 were more lethal to SPBL than high doses of SAHA (Fig 1C and 1D).

### AN-7 has a more rapid and longer lasting toxic and apoptotic effect on MF/SS cell lines than SAHA and induces stronger apoptosis in SPBL

**Fig 2:**
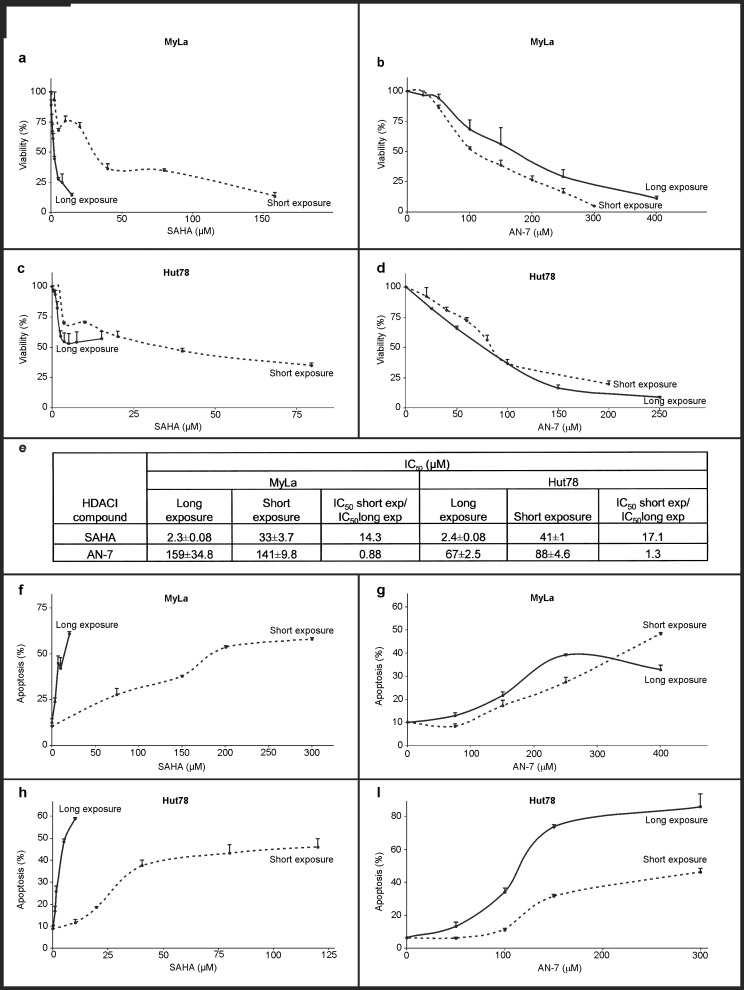
Toxic and apoptotic effect of SAHA and AN-7 on MF/SS cell lines as a function of exposure time. Viability curves based on trypan blue staining of MyLa and Hut78 cells following short or long exposure to SAHA (a, c) or AN-7 (b, d). IC_50_ values of short and long exposure to SAHA and AN-7 in MF/SS cell lines based on viability curves a-d (e). Apoptosis curves based on FACS analysis of annexin V and PI staining (f-i). Percent of apoptotic MyLa cells (early + late apoptosis) after short or long exposure to SAHA (f) or AN-7 (g), and apoptotic Hut78 cells after short or continuous exposure to SAHA (h) or AN-7 (i).

We tested the sensitivity of the MF/SS cell lines to AN-7 and SAHA after long or short exposure using trypan blue staining (Fig 2A–2E). In MyLa cells treated with SAHA, the IC_50_ of short exposure was 14.3-fold higher than the IC_50_ of long exposure (*p* = 0.0012); in Hut78 cells, the IC_50_ of short exposure was 17.1-fold higher than for long exposure (*p* = 2.28x10^-6^). By contrast, there was no difference in AN-7 toxicity in MyLa cells by length of exposure (*p* = 0.644), and only a minor difference (0.88-fold) in Hut78 cells (*p* = 0.017).

To test the apoptotic effect of the two HDACIs by length of exposure, different lengths of exposure were used and analyzed for their annexin V and PI staining. Both AN-7 and SAHA induced apoptosis in the MF/SS cell lines (Fig 2F–2I). For AN-7, there was no significant difference in the degree of apoptosis by time of exposure in either cell line (MyLa *p* = 0.9, Hut78 *p* = 0.25). However, for SAHA, short exposure was associated with 2.75-fold less apoptosis in MyLa cells (*p* = 0.034) and 2.5-fold less apoptosis in Hut78 cells (*p* = 0.046) compared to long exposure.

**Fig 3:**
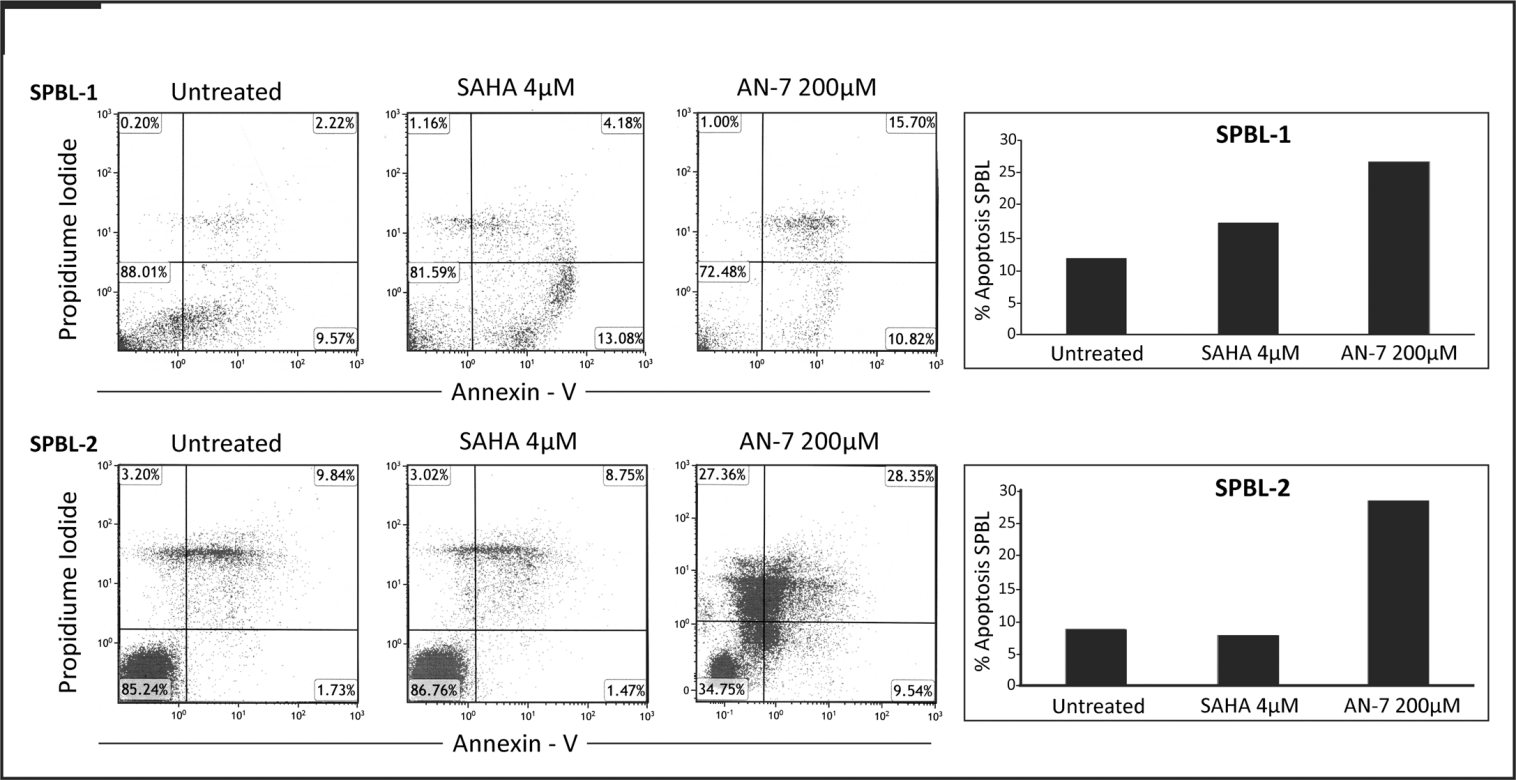
Apoptosis induction of AN-7 and SAHA in SPBL. PBL from 2 SS patients were plated at a concentration of 0.5x10^6^ cells/mL, and were then treated with SAHA 4 μM or AN-7 200 μM for 48 h. The cells were then stained with annexin V and PI. FACS plots are shown with percent of cells in each quadruplet, and the percent of cells in apoptotic cells (early + late apoptosis) are shown also in column.

Subsequently, we tested *ex vivo* the apoptosis induction of AN-7 and SAHA on two PBL samples of SS patients. The drugs’ concentrations for apoptosis induction were determined based on the average IC_50_ of each drug in SPBL (for AN-7, 128 μM and for SAHA, 2.96 μM, Fig 1E), using doses of about 1.5 folds higher than the IC_50_’s for incubation of 48 h instead of 72 h that were used in the viability assay. AN-7 directed more SPBL cells into apoptotic death than SAHA, and in one SPBL sample it induced also stronger necrosis (Fig 3).

### AN-7 and SAHA induce the expression of proapoptotic proteins, downregulate HDAC1 expression and upregulate acetylation of histone 3 (H3) in MF/SS cell lines

**Fig 4:**
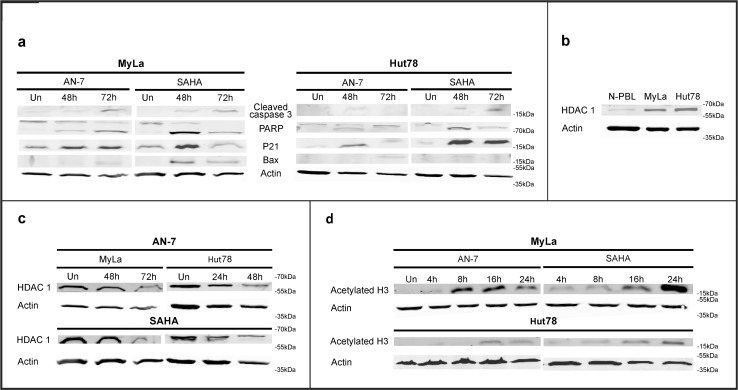
Effect of SAHA and AN-7 on specific protein expression and modification in MF/SS cell lines. Immunoblot of apoptotic and proapoptotic proteins in MyLa and Hut78 cells treated with SAHA 10 μM or AN-7 300 μM for the indicated periods (a). Basal HDAC1 protein expression in NPBL and MF/SS cell lines (b) and in MF/SS cell lines treated with SAHA 10 μM or AN-7 300 μM (c). Acetylated H3 in the nuclear lysate of MF/SS cell lines treated with and the same concentrations of SAHA and AN-7 for the indicated periods (d).

To characterize the mechanism underlying HDACI-induced apoptosis, we used Western blot analysis to measure the levels of several proapoptotic proteins in MF/SS cell lines treated with AN-7 or SAHA at concentrations previously shown to cause about 60% apoptosis (Fig 4A). Both SAHA and AN-7 treatment led to cleavage of caspase 3 and poly ADP-ribose polymerase (PARP) and the production of P21 and Bax in both cell lines. However, there was a stronger signal in response to SAHA.

Studies have shown that neoplasia, including lymphoid and myeloid leukemia, is associated with abnormalities in the expression, function, or recruitment of HDAC and/or its counterpart, histone acetyl transferase (HAT) [35]. We found that both MF/SS cell lines expressed high levels of HDAC1 compared to NPBL (Fig 4B) and that these levels were downregulated on exposure to either AN-7 or SAHA (Fig 4C). The expression of acetylated H3_,_ a direct substrate of HDACIs, was induced by both drugs, with earlier AN-7-mediated kinetics in MyLa cells (Fig 4D).

### AN-7 acts synergistically with Dox and SAHA acts antagonistically with Dox in MF/SS cell lines and SPBL

**Fig 5:**
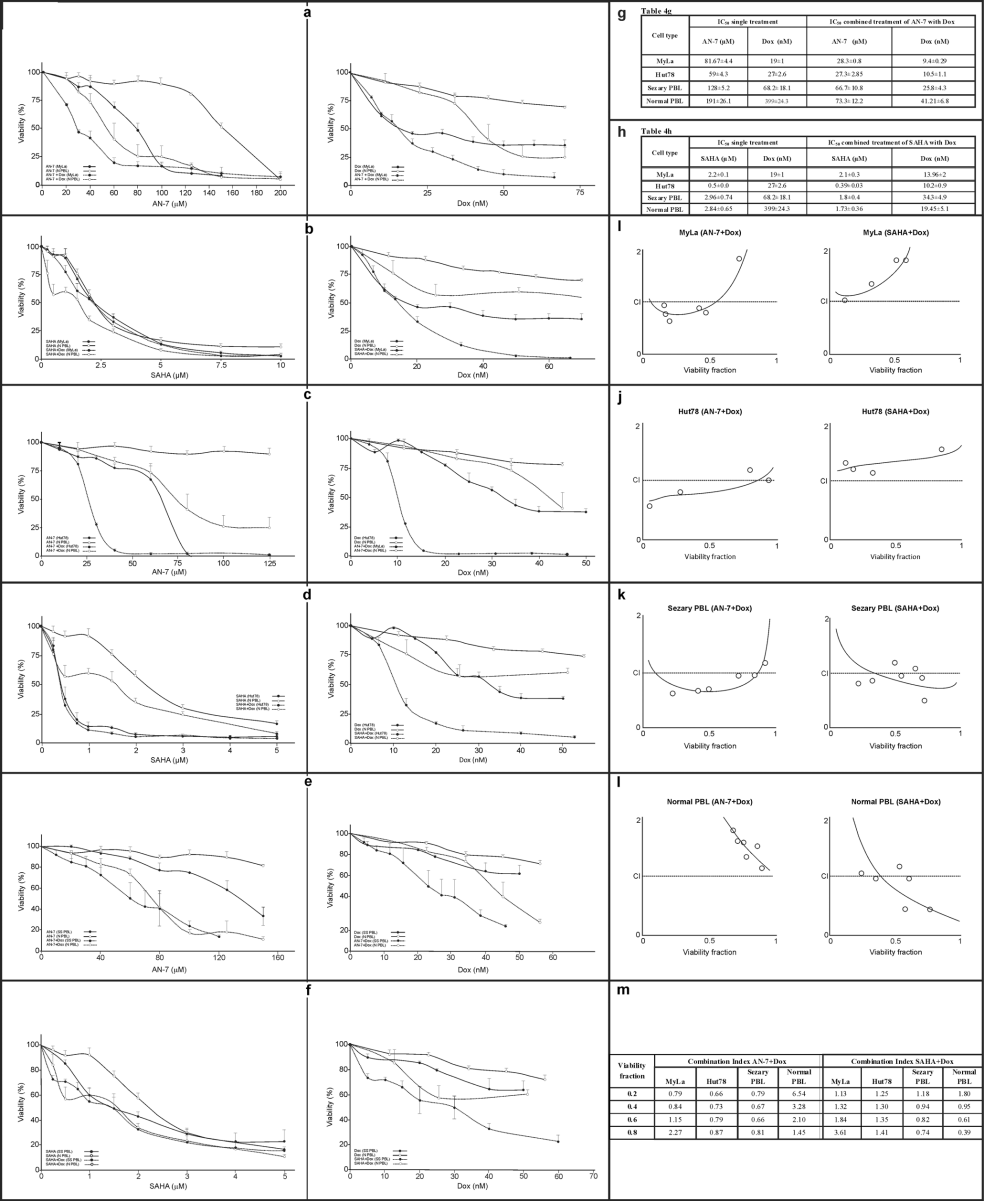
Toxicity of Dox+AN-7 and Dox+SAHA in MF/SS cell lines SPBL and NPBL. Viability curves based on the MTT assay of MyLa cells, Hut78 cells, and SPBL treated for 72 h with drug combinations, in comparison to NPBL. The combination ratio between the HDACIs and Dox were based on the ratio between their IC_50_ values for each cell type, as follows: MyLa cells treated with Dox+AN-7, 1:3000 (molar ratio) (a) and with Dox+SAHA, 1:150 (b); Hut78 cells treated with Dox+AN-7, 1:2600 (c) and Dox+SAHA, 1:38 (d); SPBL treated with Dox+AN-7, 1:1781 (e) and Dox+SAHA, 1:20 (f). NPBL were treated at same molar ratio as SPBL (a-f). Tables show IC_50_ values of AN-7 (g), SAHA (h) their combination with Dox, and Dox alone, in MF/SS cells, SPBL, and NPBL, derived from viability curves a-f. Also shown are CI-viability fraction plots of combined treatment based on viability curves a-f in MyLa cells (i), Hut78 cells (j), SPBL (k), and NPBL (l), and a table of the CIs at representative viable fractions in each cell type, derived from curves i-l (m).

MTT viability assay analysis of the anti-cancer effect and selectivity of HDACIs combined with Dox in MF/SS cell lines and SPBL compared to NPBL (Fig 5A–5H) revealed a dramatic reduction in the IC_50_ of each drug in the AN-7+Dox combination (*p* = 0.0002 in MyLa cells, *p* = 0.003 in Hut78 cells, *p* = 0.054 in SPBL) but not in the SAHA+Dox combination (*p* = 0.8, *p* = 0.3, and *p* = 0.424, respectively). In addition, the IC_50_ of AN-7+Dox exhibited strong selectivity in MF/SS cell lines compared to NPBL (MyLa *p* = 0.02, Hut78 *p* = 0.003), whereas the IC_50_ of SAHA+Dox exhibited selectivity in Hut78 cells (*p* = 0.02) and not in MyLa cells (*p* = 0.5) (Fig 5G and 5H). Differences in selectivity of the combined treatment between SPBL and NPBL failed to reach statistical significance because of the small size of the patients group. The CI-vs.-viability fraction plots demonstrated a synergistic effect of AN-7+Dox in Hut78 cells (Fig 5K) as well as in SPBL (Fig 5L). The dose combination of AN-7+Dox leaving less than 50% of viable MyLa cells was also synergistic (Fig 5I), as opposed to the antagonistic effect in NPBL (Fig 5M). The CI- vs.-viability fraction plots demonstrated an antagonistic effect of SAHA+Dox in both MyLa and Hut78 cell lines (Fig 5I and 5K). The dose combination of SAHA+Dox leaving less than 50% of viable SPBL had an antagonist-to-additive effect, with similar results in NPBL (Figs 5L and 4M respectively).

## Discussion

This study shows that AN-7 is an effective drug against MF/SS. It acts synergistically with Dox and has some advantages over SAHA *in vitro* and *ex vivo*. In this section, we define the nature of the relative efficacy of AN-7 and on this basis, discuss its potential for the clinical treatment of MF/SS.

HDACIs preferentially kill transformed cells, whereas normal cells are relatively resistant [11,19]. AN-7 showed greater selectivity than SAHA against MyLa cells, Hut78 cells, and SPBL due to the higher resistance of normal cells to AN-7 and their lower resistance to SAHA. The different HDAC targets may explain the difference in anti-cancer selectivity of AN-7 and SAHA. For instance, HDAC6 is inhibited by SAHA but not by AN-7 [36,37]. The lesser sensitivity of MyLa cells than Hut78 cells to AN-7 and SAHA may be attributable to the twofold higher proliferation rate of Hut78 cells. The *ex vivo* advantage of AN-7 over SAHA as a selective anticancer drug might prove to be clinically relevant for patients with SS.

Our apoptosis and viability assays demonstrated that AN-7 works faster than SAHA and is highly effective and selective after both short and continuous treatment. For SAHA to achieve a maximum apoptotic effect, it needed to be present for a longer time than AN-7. Whether the differential kinetics of the two drugs confers clinical superiority to AN-7 over SAHA needs to be tested in a clinical trial. We found that a similar percent of Hut-78 cells undergo apoptosis after exposure to both AN-7 and SAHA, while AN-7 induces more apoptosis in SPBL. In MyLa cells, SAHA promotes more apoptosis than AN-7, but it requires continuous exposure. Moreover, we have shown that AN-7 as well as SAHA induces cleavage of Caspase-3 to its activated cleaved form, which leads to cleavage of the apoptosis hallmark protein-PARP, followed by up regulation of p21 and Bax. The apoptotic signal measured by those apoptotic and proapoptotic proteins was slightly stronger in cells treated with SAHA than AN-7. However, both induce the same percent of apoptosis (about 60%). Therefore, we assume that only large *ex vivo* study in PBL of SS and lymphoma cells from MF lesions of patients will determine which of these HDACIs has stronger apoptotic potential for *in vivo* relevancy.

Aberrant regulation of apoptosis is a central pathological feature of MF and SS and other lymphoma types, and it correlates with more aggressive disease and resistance to Fas-mediated apoptosis [38–40]. The expression of upstream Fas pathway factors, Fas, Fas ligand, and the FLICE-Fas like inhibitory protein in MF lesions were found to support the defective apoptosis in MF [39]. Accordingly, the most successful CTCL therapies involve the induction of T-cell apoptosis [40]. Studies of the role of HDACIs in MF and SS reported increased levels of the death receptor ligands FasL and TRAIL in the extrinsic pathway in transformed cells but not in normal cells [41].

The elevated level of acetylated H3 observed in this study after exposure of MF/SS cell lines to AN-7 confirmed its function as an HDACI. The reduction in the expression of HDAC1 by AN-7 was already demonstrated in other cancer cell lines as well [28]. In MyLa cells, acetylated H3 accumulated earlier on exposure to AN-7 than to SAHA, suggesting a more rapid action of AN-7 in inhibiting HDAC activity. The level of HDAC classes I and II vary in different cancer cells [42]. We show that MF/SS cell lines highly express HDAC1 relative to their normal counterpart, PBL, which was downregulated by both SAHA and AN-7, with late kinetics. Thus, SAHA and AN-7 apparently inhibit the activity of HDAC enzymes and thereafter influence their expression level resulting in prolonged acetylation of histone and non-histone proteins. Whether the differential kinetics of AN-7 also confers clinical superiority over SAHA needs to be tested in a clinical trial.

Molecularly targeted drugs and cytotoxic chemotherapy are associated with *de novo* and acquired resistance, limiting their utility [43]. The effectiveness of chemotherapy may be improved by drug combinations aimed at targeting multiple pathologic processes, lowering drug doses, minimizing adverse side effects, and reducing disease recurrence [43, 44]. Studies have reported a synergistic effect of SAHA with N-(4-hydroxyphenyl) retinamide (fenretinide), or the proteosome inhibitor bortezomib in leukemia cells [45], primary effusion lymphoma cell lines [46], and rhabdoid tumors in animal models [47]. In addition, a recent study found that the combination of UV_A_Sens/UV-A photochemotherapy with the HDACIs SAHA and MS-275 significantly decreased cell viability and increased apoptosis and DNA-double strand breaks in MyLa cells [48].

Chemotherapy plays a major part in cancer management; however, along with benefits to patients it causes severe adverse effects [49]. Dox is one of most effective drugs currently available for the treatment of neoplastic diseases. However, its use is complicated by dose-limiting cardiotoxicity and therefore requires combination therapy [50]. The synergistic interaction between AN-7 and Dox was reported in several cancer cell lines and animal models [26, 29]. In addition to the synergy, AN-7 was shown to protect animals against cardiotoxicity induced by Dox, which add an important benefit to the quality of life for the patients.

A synergistic interaction between HDACI and Dox has been reported in several cancer cell lines [19,20, 51]. The present study is the first to combine SAHA or AN-7 with Dox in MF/SS cell lines and SPBL. We found that AN-7 and Dox interact synergistically in MF/SS cell lines and SPBL, reducing their viability, and antagonistically in NPBL. Although previous studies, which reported a synergistic effect of SAHA with Dox in other malignant cell lines [47, 52, 53], in MF/SS cell lines, we found that SAHA and Dox interacted antagonistically, at drug doses leaving less than 50% of viable cells. Similar findings were noted for SPBL and NPBL. Taken together, these results provide a rationale for combining Dox with AN-7, but not with SAHA, to induce selective death of MF/SS cells.

The ability of anthracyclines to kill cells is correlated with their ability to induce DNA adducts [21]. Studies of human carcinoma cells showed that the quantity of Dox-DNA adduct formation was negligible in the absence of AN-7 and increased dramatically when AN-7 was added [26]. Combining HDACIs with agents that induce DNA damage leads to a sustained DNA-damage response coupled with insufficient repair, with particular effectiveness in cancer cell apoptosis [19,20,54,55]. It has previously been shown that mice injected with human mammary cancer cell line followed by treatment with AN-7+Dox showed enhanced DNA damage, and reduced angiogenesis, tumor growth, and metastasis compared to mice treated with Dox only [29].

## Conclusions

The present study shows that AN-7, a novel HDACI, holds promise for the treatment of MF/SS. Although SAHA, the first HDACI approved for the treatment of CTCL, exhibits stronger signals of apoptotic markers in MF/SS cell lines, and induces more apoptosis in MyLa cells, yet, AN-7 induces more apoptosis in PBL from patients with SS, and affects MF/SS cell lines more rapidly, for a longer time, and with better selectivity. Moreover, AN-7 also showed selectivity to PBL of SS patients, whereas SAHA did not. The clinical implications of these advantages of AN-7 over SAHA, namely, a shorter time to response and better safety profile, warrant further investigations. Furthermore, AN-7 interacts synergistically and selectively with Dox to kill MF/SS cell lines and SPBL, but SAHA interacts antagonistically with Dox. These data provide a rationale for combining AN-7, but not SAHA, with Dox for the treatment of patients with MF/SS.
